# Orthodontic Adolescent Patients’ Attitudes toward Protective Face Mask Wearing during the COVID-19 Pandemic

**DOI:** 10.3390/medicina58030393

**Published:** 2022-03-06

**Authors:** Jessica Olivia Cherecheș, Luminița Ligia Vaida, Abel Emanuel Moca, Raluca Dima, Gabriela Ciavoi, Marius Bembea

**Affiliations:** 1 Department of Dentistry, Faculty of Medicine and Pharmacy, University of Oradea, 10 Piața 1 Decembrie Street, 410073 Oradea, Romania; chereches_jessica@yahoo.com (J.O.C.); razdima@gmail.com (R.D.); gciavoi@uoradea.ro (G.C.); 2 Department of Preclinical Disciplines, Faculty of Medicine and Pharmacy, University of Oradea, 10 Piața 1 Decembrie Street, 410073 Oradea, Romania; bembea13@yahoo.com

**Keywords:** COVID-19, protective face mask, orthodontic patients, adolescents

## Abstract

*Background and Objectives:* The COVID-19 pandemic led to restrictive measures, which aimed to limit the spread of the SARS-CoV-2 virus. These restrictions impacted all areas of life, including the activity of dental offices. For patients with orthodontic appliances, closing the dental offices was a major issue, as most orthodontic treatments last for more than a year and require regular checkups. The aim of this research was to assess the impact that the restrictive measures that were imposed during the COVID-19 pandemic, and, especially, wearing a face mask had on a sample of Romanian teenagers undergoing fixed orthodontic treatment. *Material and Methods:* The study group consisted of 277 orthodontic patients, with ages between 12 and 17.9 years, from North-Western Romania. They completed a 9-item questionnaire. The control group consisted of 231 participants, with ages between 12 and 17.9 years. They completed an 8-item questionnaire. *Results:* Most patients from the study group were not worried that wearing a protective face mask would hide their braces (never—49.5%; rarely—26.7%), and their desire to undergo an orthodontic treatment was not affected by the compulsoriness of face mask wearing (never—51.6%; rarely—26%). In contrast to that, in the control group, more than 50% of the participants were worried to some degree that wearing a protective face mask would hide their smile (occasionally—29.9%; frequently—18.2%; very frequently—2.2%). The majority of the participants from the study group did not consider interrupting the orthodontic treatment due to the COVID-19 pandemic (62.5%), and the majority of the participants from the control group did not consider not going to the dentist due to the COVID-19 pandemic (70.6%). Most of the participants from the study group were not happy that they had to wear a face mask, which covered their orthodontic appliances, during the orthodontic treatment (68.6%). The attitude was similar to that of the participants from the control group, who were not happy that they had to wear a face mask, that covered their smile (51.1%). In the study group, most patients did not want face mask wearing to continue to be compulsory, given the fact that their orthodontic appliances were no longer visible (52%). In the control group, the attitude was similar, with 48.1% of the participants not wanting face mask compulsoriness to be maintained. *Conclusions*: In conclusion, although, most patients would not like to continue wearing a face mask as a mandatory regulation, they were not concerned or negatively affected by wearing a protective face mask, even though face masks hid their braces.

## 1. Introduction

The identification of a new type of coronavirus at the end of 2019, outlined the scenario that announced the beginning of a major public health crisis worldwide [1]. The disease, a form of severe acute respiratory syndrome, caused by SARS-CoV-2 and called COVID-19, has been described by the World Health Organization as being a viral pneumonia [2]. The symptoms are numerous, and include dry cough, fever, shortness of breath, sore throat, headache, myalgia, fatigue, diarrhea [3], and radiological signs of lung damage [4].

The transmission rate of COVID-19 is high, as it spreads easily from person to person [5]. Coughing, sneezing, or talking can generate aerosols [6], which through close contact with infected people are safe sources of contamination [5]. The virus can enter the body by air and attaches to the mucous membranes of the oral cavity, nose, or eyes [6,7].

As a result of the increase in the number of cases with COVID-19 disease, a pandemic was declared, and worldwide, in an attempt to limit the spread of the virus, quarantine was instated in all countries [8]. Among other restrictions, a safe distance of 1–2 m had to be maintained between people [9]. For dental professionals, this distance was impossible to maintain, as dental work requires close contact with the patient’s oropharyngeal and nasal region, increasing the risk of contamination [9,10]. The activity of dental offices was suspended, which affected all patients [11]. Emergency dental treatments consisting of pain, swelling, bleeding, infections, and trauma were permitted in certain authorized dental offices [12,13].

For patients with orthodontic appliances, closing the dental offices was a major issue, as most orthodontic treatments last for more than a year [12], and require regular checkups [14]. During this period of disruption, treatments could no longer be supervised and were negatively affected, with patients reaching high levels of anxiety caused by the new situation [12]. 

Face mask wearing became mandatory in many countries as it is considered an important preventive measure during the COVID-19 pandemic [15]. Face masks are considered beneficial since wearing a mask in areas where the advised social distance cannot be properly maintained lowers the spread of virus-loaded droplets [16]. As they cover the nose and mouth of the patients, and the area around them [17], protective face masks also cover the fixed orthodontic appliances bonded on the buccal surface of the teeth, which are visible in smile and speech. For some patients this aspect could potentially cause frustration, since the desire for orthodontic treatment among teenagers has been proven to be high [18], and patients are usually satisfied with their facial aspect during the orthodontic treatment [19]. The question was raised whether the necessity to cover the orthodontic appliance with a face mask could cause teenagers to be less interested in undergoing a necessary orthodontic treatment, especially because often braces are perceived as being an elective luxury and a symbol of status, wealth, and style [20], or whether it could nurture a disobedience towards the mandatory wearing of face masks.

The aim of this research was to assess the attitude that a sample of Romanian teenagers undergoing orthodontic treatment with fixed appliances, during the COVID-19 pandemic, had regarding wearing protective face masks, considering the fact that they covered the orthodontic appliances. Their attitude towards the suspension of dental activity, as orthodontic patients, was investigated as well.

## 2. Materials and Methods

### 2.1. Ethical Considerations

The study was conducted in accordance with the 1964 Declaration of Helsinki and its later amendments and was approved by the Research Ethics Committee of the University of Oradea (No. 23/25.02.2021). Before filling in the questionnaires, all parents, caregivers, and participants gave their consent for taking part in this study.

### 2.2. Sample Size Calculation

Sample size estimation was made using GPower 3.1.9.7 software. By the design of the study, it was considered that the measured items (in Likert scale format) would be mostly compared between genders, using Mann–Whitney U tests (for items with 5 answers) or contingency tables (for items with 3 answers), and the ideal allocation ratio of the genders should be 1:1. Therefore, it was estimated using a medium effect size of d = 0.5, with a minimum power of 0.8 and an α = 0.05, that the minimum sample size should be of 74 patients in each group for Mann–Whitney U tests (a total of 168). For contingency tables, considering a medium effect size of w = 0.3 with Df = 2, a minimum power of 0.8 and an α = 0.05, the minimum total sample size should be equal to 108. Using these values, an estimation was made that a minimum of 74 patients in each gender (with a total of 168 patients) should exist in the study for a minimum power of 0.8 for most tests.

### 2.3. Participants and Data Collection

The study design was a cross-sectional survey. It was carried out in the period between November 2020 and February 2022. A pilot study was not conducted prior to this research. During the period in which this study was conducted, restrictive measures regarding the mandatory wearing of face masks and social distancing were active. 

For the study group, the authors designed a questionnaire consisting of 9 items. For the control group, only 8 items were used, since Item 9 referred strictly to orthodontic patients. The questionnaires were printed on paper and applied in two private orthodontic practices from the city of Oradea, North-Western Romania, which offer treatments to patients who come from families with various incomes (from low to high). They were distributed to adolescents, aged between 12 and 17.9 years. In the study group, the respondents were orthodontic patients, undergoing an orthodontic treatment with fixed appliances. In the control group, the respondents were non-orthodontic patients, who came to the office for a clinical examination. Before filling in the questionnaire, all patients and their parents (or caregivers) were informed that they were being applied for research purposes, and that by filling in the questionnaires, they confirmed their willingness to participate anonymously in this study. The names of the participants were not mentioned on the survey form, and the authors did not know how patients answered. Patients had the possibility to withdraw from the research with no consequences, and no financial incentives were promised to the respondents. No time limit was imposed. The language used for the questionnaires was Romanian.

A Likert-type scale was used for Items 1, 2, 3, 4, and 8. The options included were “never”, “rarely”, “occasionally”, “frequently”, and “very frequently”. For Items 5, 6, 7, and 9, participants had to choose from three options, these being “no”, “yes”, and “maybe”. Items are translated and detailed in Table 1.

For the study group, the inclusion criteria were that the participants had to be patients wearing a metallic or ceramic fixed orthodontic appliance (bonded on the buccal surface of teeth, and visible in smile and speech), with ages between 12 and 17.9 years, and to live in Romania. The control group consisted of participants who were not wearing and did not wear orthodontic appliances (fixed or removable), with ages between 12 and 17.9 years, and were living in Romania. Patients who were in the contention phase of the orthodontic treatment, as well as questionnaires that were incomplete or incorrectly completed were excluded from this study. Incorrectly completed questionnaires were those where the patients offered more than one answer for the same item. 

### 2.4. Statistical Analysis

Statistical analysis was performed by using IBM SPSS software, version 25 (IBM, Chicago, IL, USA). Quantitative variables were tested for distribution using the Shapiro–Wilk test and were expressed as mean values with standard deviations or medians with interpercentile intervals. The independent quantitative variables with a non-parametric distribution were tested with the Mann–Whitney U or Kruskal–Wallis H tests, and all correlations between them were verified with Spearman’s rho correlation coefficient. Qualitative variables were expressed as absolute numbers or percentages and were tested with Fisher’s exact test. 

## 3. Results

**Figure 1:**
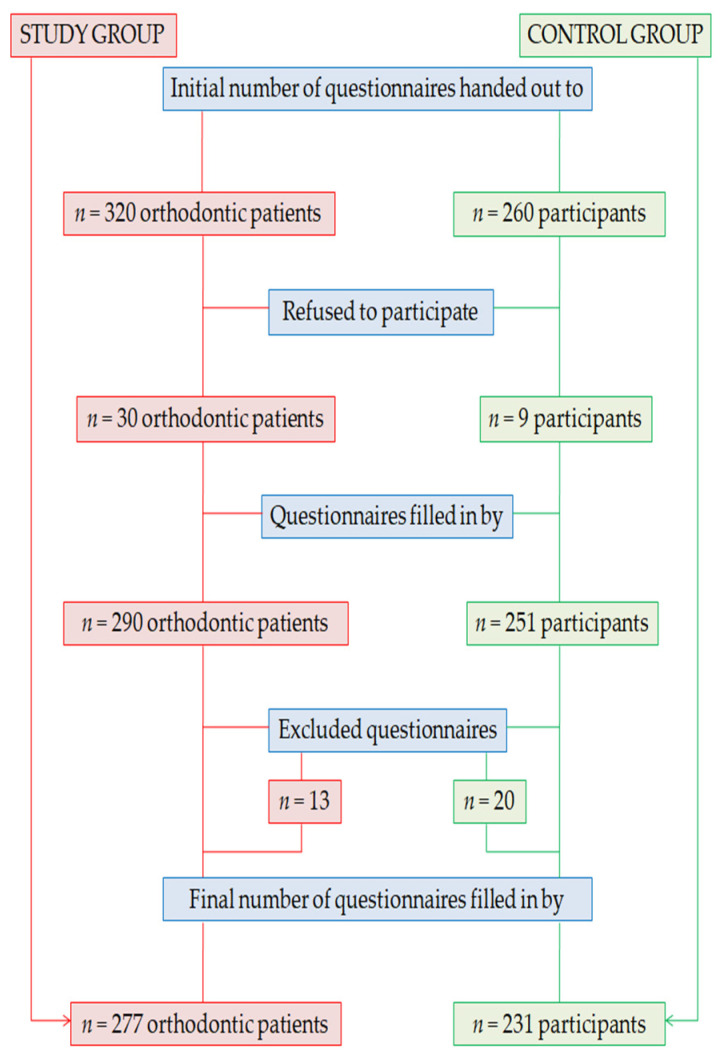
Study flowchart.

In the study group, the questionnaires were handed out to 320 orthodontic patients, but only 290 agreed to take part in this research and filled in the survey forms. After applying the exclusion criteria, 277 valid questionnaires remained in the study. In the control group, the questionnaires were handed out to 260 participants, but only 251 agreed to take part in this research and filled in the survey forms. After applying the exclusion criteria, 231 valid questionnaires remained in the study (Figure 1).

### 3.1. Socio-Demographic Data

The study group consisted of 173 (62.5%) girls and 104 (37.5%) boys. Regarding the living environment of the participants, 93 (33.6%) came from a rural environment, while 184 (66.5%) came from an urban environment. The mean age of the respondents was 14.91 ± 1.49 years, with a median of 15 years, and a range between 12 and 17.9 years.

The control group consisted of 134 (58%) girls and 97 (42%) boys. Regarding the living environment of the participants, 92 (39.8%) came from a rural environment, while 139 (60.2%) came from an urban environment. The mean age of the respondents was 14.77 ± 1.64 years, with a median of 15 years and a range between 12 and 17.9 years.

Data in Table 2 shows the comparison of participants’ ages in relation to the living environment. According to the Shapiro–Wilk test, in the study sample, age distribution was non-parametric in both groups, and the Mann–Whitney U test showed that age differences were significant. Participants living in an urban environment had a higher age than those living in a rural environment. In the control sample the age difference was not significant between the groups. 

The Mann–Whitney U test showed that the age of the participants was not statistically significant between the study group and the control group (*p* = 0.346). The living environment of the participants was, also, not statistically significant between the study group and the control group (*p* = 0.165).

### 3.2. Attitude towards Protective Face Mask Wearing and Treatment Interruption

Data in Table 3 shows the distribution of the patients according to the answers given for all 9 items (study group) and 8 items (control group). In the study group, most patients were not worried that wearing a protective face mask would hide their braces (Item 1), and their desire to undergo an orthodontic treatment was not affected by the compulsoriness of face mask wearing (Item 2). The majority of the participants did not consider interrupting the orthodontic treatment due to the COVID-19 pandemic (Item 5), but most of them were not happy that they had to wear a face mask, which covered their orthodontic appliances, during the orthodontic treatment (Item 6), and did not want face mask wearing to continue to be compulsory, given the fact that their orthodontic appliances were no longer visible (Item 7). In the control group, more than half of the participants were worried to some degree (occasionally, frequently, very frequently) that wearing a protective face mask would hide their smile (Item 1) and were affected by the compulsoriness of face mask wearing (Item 2). The majority of the participants did not consider not going to the dentist due to the COVID-19 pandemic (Item 5).

### 3.3. Correlational Results

In the study group, statistically significant correlations were found between respondents’ age and answers provided for Items 1, 3 and 8. As such, patients with higher ages were less concerned about the fact that wearing a protective face mask would hide their braces and were less affected by the suspension of dental offices’ activity, as patients undergoing an orthodontic treatment with fixed appliances. However, younger patients were less stressed about wearing a protective face mask that hid the orthodontic appliances (Table 4). In the control group, there were no statistically significant correlations identified between respondents’ age and answers provided for Items 1, 3, and 8.

In the study group, patients’ gender influenced the answers received for Items 6 and 7. As such, boys were unhappier with wearing a protective face mask during the orthodontic treatment, while indecisive patients were more frequently girls. Boys were, also, less eager to continue wearing a face mask as a mandatory regulation, considering the fact that face masks covered the braces. The answers received for Items 5, 7, and 9 were significantly influenced by participants’ living environment. Patients living in an urban environment were less prone to considering interrupting the orthodontic treatment as a result of the COVID-19 pandemic, and were more eager to maintain the compulsoriness of face mask wearing, even though face masks covered the orthodontic appliances. Patients living in a rural environment were more frequently indecisive about interrupting the orthodontic treatment during the COVID-19 pandemic, were less eager to maintain the compulsoriness of face mask wearing during the orthodontic treatment, and were more frequently indecisive about interrupting the orthodontic treatment while wearing a face mask (Table 5). In the control group, only the answers received for Item 7 were influenced by patients’ gender. As such, girls were unhappier than boys about the fact that they have to wear a protective face mask that would cover their smile. The answers received for Item 5 were significantly influenced by participants’ living environment. As such, patients living in a rural environment were more frequently indecisive than patients living in an urban environment about not going to the dentist due to the COVID-19 pandemic (Table 5).

Other significant correlations were found between answers provided for some items. As such, in the study group, participants who were more concerned that wearing a face mask would hide their orthodontic appliance (Item 1) considered that the compulsoriness of face mask wearing affected their desire to undergo an orthodontic treatment, because face masks covered their braces (Item 2) (*p* < 0.001, R = 0.300), and were more affected by the suspension of dental offices’ activity, as patients undergoing an orthodontic treatment with fixed orthodontic appliances (Item 3) (*p* = 0.001, R = 0.194). 

Patients who did not want protective face masks to continue being mandatory, given the fact that they covered the braces (Item 7) were more frequently indecisive about their willingness to continue the orthodontic treatment while wearing a protective face mask (Item 9) (Table 6).

### 3.4. Comparative Results

Comparisons were made between participants considering their age, gender, and living environment. Although significant differences were found for some items in relation to age and gender, no significant differences were found in relation to the living environment. Regarding age, in the study group, significant differences were found for Item 9. A comparison of age in relation to patients’ desire to continue the orthodontic treatment while wearing a face mask was made. Age distribution was non-parametric in most groups according to the Shapiro–Wilk test (*p* < 0.05). The differences between groups were statistically significant according to the Kruskal–Wallis H test (*p* = 0.002), and post-hoc tests showed that indecisive patients had a lower age than patients who said they do not want to continue the orthodontic treatment while wearing a face mask, given the fact that the orthodontic appliances were no longer visible (*p* = 0.044) or than patients who said they want to continue the orthodontic treatment while wearing a face mask, despite the fact that the orthodontic appliances were no longer visible (*p* = 0.001).

Regarding patients’ gender, in the study group significant differences were identified for Items 2 and 4. The results obtained showed that boys’ desires to undergo orthodontic treatment were less affected by the compulsoriness of a face mask that covers the braces, in comparison with girls, and they were less worried about the possibility of the orthodontic treatment suspension than girls (Table 7). In the control group, significant differences were identified for Item 4. The results were similar to the study group. Girls were more worried that they would not be able to go to the dentist due to the COVID-19 pandemic (Table 7). 

Other significant differences were found between answers provided for some items. In the study group, significant differences were identified between Items 1 and 9, Items 2 and 5, Items 3 and 5, and Items 3 and 7. In the control group, significant differences were identified between Items 3 and 5 and Items 3 and 7. They are detailed in Table 8, which shows the results of the Kruskal–Wallis H test and Shapiro–Wilk test. 

Table 9 and Table 10 show the comparisons of answers provided for Item 1 to Item 8, according to the analyzed groups (study group and control group). Statistically significant differences were identified for most items. As such, participants undergoing an orthodontic treatment with fixed appliances (study group) were less affected by the compulsoriness of face mask wearing, in comparison to the participants from the control group (Item 2) and were worried that they would not be able to continue the orthodontic treatment (Item 4). In comparison with the study group, participants from the control group were more worried about the fact that they had to wear a protective face mask (Item 1) and were more stressed that they had to wear a protective face mask (Item 8).

## 4. Discussion

The COVID-19 pandemic has had a strong impact on the global healthcare system, affecting both the economy of health systems [21] and the way patients have accessed healthcare services. During the pandemic, the use of healthcare fell by about a third among patients [22]. Dental practices have also been affected by the restrictions imposed during the pandemic. Restrictive measures maintained for a longer period of time could lead to financial distress, the most affected being dental practices with high operational costs [23]. Withholding dental care led to the progression of undiagnosed and untreated oral diseases [24], and orthodontic treatments were suspended during the lockdown period, with orthodontists being unable to sustain ongoing treatments. In Romania, a state of lockdown was established for a period of two months, between March 16 and May 16, 2020 [25]. 

It is important to determine patients’ attitudes towards restrictive measures and, especially, towards wearing a protective mask, during the orthodontic treatment, keeping in mind that face masks cover the orthodontic appliances. For this purpose, we designed a short questionnaire, comprising only 9 items (8 items for the control group), to which patients can easily answer in the waiting room, before completing their regular checkup. The use of questionnaires is an accessible method for collecting data from patients, in order to conduct a statistical study [26]. Although most research in the field of dentistry investigating different attitudes during the COVID-19 pandemic is based on questionnaires applied on online platforms [27,28], websites [29], e-mail addresses of dentists [30] or patients, or both online and on paper [31], in the present study the paper printed version of the questionnaire was used for a better selection of patients and for an easier application of the inclusion and exclusion criteria. Additionally, by completing the questionnaires in the dental office after a longer period of wearing a face mask, patients were given clarifications about any questions they could not comprehend. However, completing the questionnaires in the dental office could make participants feel obligated to respond in a manner that would positively represent an orthodontic treatment. In order to reduce this possibility, patients were encouraged to answer freely and honestly, and were assured that the questionnaires will remain anonymous.

Although there are other studies that have examined the impact of the pandemic on orthodontic patients, they have attempted to determine the challenges that patients had and the solutions proposed by them [1]. Our study focused mainly on finding out information about the patients’ attitudes towards wearing a protective mask during the orthodontic treatment. We selected adolescent patients because, generally, they are the main population group receiving orthodontic treatment [32]. The protective mask covers the middle and lower third of the face, thus covering the teeth in smile and speech. In this way, the orthodontic appliance is no longer visible. In our study, although the desire of most patients to undergo the orthodontic treatment was not negatively affected by wearing a face mask, even though face masks covered their braces, most patients were not happy to wear a face mask while wearing the orthodontic appliance. In our view, a potential reason for the unhappiness caused by face mask wearing during the orthodontic treatment could be that adolescent patients, for the most part, want to have the orthodontic appliance visible in smile and speech, since they can be viewed as a fashion statement [20]. A type of fixed braces called “fashion braces” has been developed, with the unique purpose of imitating an orthodontic treatment, but having no therapeutic effect [20]. In other studies, however, there is a preference of young patients for less visible appliances, such as clear aligners [33], while adult patients prefer aesthetic orthodontic appliances [34]. It would be beneficial to find out if face mask wearing could increase patients’ preferences towards less esthetic orthodontic appliances, given the fact that they would not be visible while wearing the protective face mask. Nonetheless, teenagers require further support and stimulation for continuing to respect the COVID-19 mandatory regulations. They should be involved in educational programs that help other people understand the benefits of face mask wearing during the COVID-19 pandemic.

Most teenagers in this study did not consider that it would be necessary to interrupt the treatment due to the COVID-19 pandemic. The lack of concern about the fact that wearing a protective face mask would cover the orthodontic appliance increased with the age of the respondents, so that patients with higher ages were less concerned about the fact that wearing a mask would cover the orthodontic appliances (*p* = 0.001, R = −0.204). This suggests that younger patients were more eager to have their orthodontic appliance visible. Generally, people are not concerned about wearing a protective face mask, and although there is an impression that the anti-mask sentiment is widespread [35], most studies show that people are willing to follow the authorities’ recommendations and wear a face mask [35].

Regarding the living environment of the respondents and their attitude towards wearing a protective mask, it can be emphasized that patients living in an urban environment were more eager to maintain the compulsoriness of face mask wearing, even though the face mask covered the orthodontic appliances, while patients living in a rural environment were less eager to maintain the compulsoriness of face mask wearing (*p* = 0.005). This may be due to the fact that the rural population engages less in preventive health behaviors than the urban population [36,37], as other studies suggested.

Regarding the sample size estimation, it is important to highlight the fact that when the sample size was estimated, an ideal gender allocation ratio of 1:1 was taken into consideration. The main goal was to determine the minimum number of questionnaires that had to be completed for each gender. However, more questionnaires than initially planned were distributed, and we did not want to lose the information obtained through these forms. We do not consider that the final distribution of patients could influence the obtained results.

The COVID-19 pandemic and the restrictions connected to it have changed the lives of patients [38]. Despite some inconveniences caused by wearing protective masks [39], wearing protective face masks should be encouraged, because they offer a high protection against the spread of the SARS-CoV-2 virus [40]. Protective face masks, along with proper ventilation, social distancing [41], and vaccination, are the safest methods to combat the COVID-19 pandemic [42]. 

The limitations of this study are related, first of all, to the number of items. It would be useful to extend the questionnaires and add more items, investigating the attitudes toward orthodontic appliances, oral health, self-esteem, and the main purpose for wearing orthodontic appliances. Extending the questionnaire to the adult population would be beneficial, since it would offer an even more comprehensive view of face mask wearing during the orthodontic treatment. The application of online questionnaires could allow a multicenter, national approach of this topic. In this way, data could be collected from several orthodontic practices from across Romania. However, given the restrictive circumstances in which this survey was conducted, during the COVID-19 pandemic, we consider it to be a solid starting point for future research. Even though mask policies are temporary, the COVID-19 pandemic is still actively causing infections [43], meaning that face masks may remain useful for an indefinite amount of time. Moreover, there are still areas at risk of impactful spillover, which could be the starting point of future pandemics [44]. Under these conditions, face masks could become the new normal.

## 5. Conclusions

Adolescents wearing fixed orthodontic appliances had a generally positive attitude towards protective face masks, despite the fact that they covered their orthodontic appliances, most of them not being bothered by the fact that face mask wearing was mandatory and not being concerned that they must wear a protective mask that would cover their orthodontic appliances. Usually, boys were less affected by the compulsoriness of face mask wearing, during the orthodontic treatment, in comparison with girls, and they were less worried about the possibility of orthodontic treatment suspension than girls. Wearing a face mask remains one of the key measures in the prevention of the SARS-CoV-2 virus spread and should be encouraged. Non-orthodontic patients were more worried about the fact that they had to wear a protective face mask and more stressed about this issue. 

## Figures and Tables

**Table 1:** Items.

	Number of Item	Question
Study group	Item 1	“Are you worried that wearing a protective face mask will hide your braces?”
	Item 2	“Does the compulsoriness of wearing a protective face mask affect your desire to undergo the orthodontic treatment, given the fact that it covers your braces?”
	Item 3	“Were you affected by the suspension of dental offices’ activity, as a patient undergoing an orthodontic treatment with fixed appliances?”
	Item 4	“Were you worried that you won’t be able to continue the orthodontic treatment due to the COVID-19 pandemic?”
	Item 5	“Did you consider interrupting the orthodontic treatment because of the COVID-19 pandemic?”
	Item 6	“Are you happy that you have to wear a face mask during the orthodontic treatment, considering the fact that it covers your braces?”
	Item 7	“Do you want face masks to continue being mandatory, given the fact that they cover your braces?”
	Item 8	“Do you consider that wearing a face mask that hides your orthodontic appliance causes you stress?”
	Item 9	“Do you still want to continue with the orthodontic treatment while wearing a face mask, even though your orthodontic appliance is not visible?”
Control group	Item 1	“Are you worried that wearing a protective face mask will hide your smile?”
	Item 2	“Does the compulsoriness of wearing a protective face mask affect you, given the fact that is covers your smile?”
	Item 3	“Were you affected by the suspension of dental offices’ activity?”
	Item 4	“Were you worried that you won’t be able to go to the dentist due to the COVID-19 pandemic?”
	Item 5	“Did you consider not going to the dentist because of the compulsoriness of face mask wearing?”
	Item 6	“Are you happy that you have to wear a face mask, considering the fact that it covers your smile?”
	Item 7	“Do you want face masks to continue to be mandatory, given the fact that they cover your smile?”
	Item 8	“Do you consider that wearing a face mask that hides your smile causes you stress?”

**Table 2:** Comparison of participants’ ages in relation to the living environment.

Living Environment	Mean Value ± SD (Years)	Median (IQR) (Years)	Medium Rank	*p* *
Study group				
Rural (*p* < 0.001 **)	14.68 ± 1.52	15 (14–15)	123.05	0.016
Urban (*p* < 0.001 **)	15.03 ± 1.47	15 (14–16)	147.06	
Control group				
Rural (*p* = 0.002 **)	14.8 ± 1.68	15 (14–16)	117.47	0.783
Urban (*p* < 0.001 **)	14.76 ± 1.61	15 (13–16)	115.03	

SD—standard deviation; IQR—interquartile range; * Mann–Whitney U test; ** Shapiro–Wilk test.

**Table 3:** Distribution of the patients according to the answers provided.

**5 Option Items**					
**Answer** **(No., %)**	**Never**	**Rarely**	**Occasionally**	**Frequently**	**Very** **Frequently**
**Study group**					
Item 1	137 (49.5%)	74 (26.7%)	21 (7.6%)	26 (9.4%)	19 (6.9%)
Item 2	143 (51.6%)	72 (26%)	29 (10.5%)	25 (9%)	8 (2.9%)
Item 3	130 (46.9%)	44 (15.9%)	33 (11.9%)	39 (14.1%)	31 (11.2%)
Item 4	66 (23.8%)	69 (24.9%)	64 (23.1%)	40 (14.4%)	38 (13.7%)
Item 8	143 (51.6%)	47 (17%)	63 (22.7%)	14 (5.1%)	10 (3.6%)
**Control group**					
Item 1	71 (30.7%)	44 (19%)	69 (29.9%)	42 (18.2%)	5 (2.2%)
Item 2	41 (17.7%)	35 (15.2%)	42 (18.2%)	92 (39.8%)	21 (9.1%)
Item 3	48 (20.8%)	99 (42.9%)	17 (7.4%)	63 (27.3%)	4 (1.7%)
Item 4	116 (50.2%)	38 (16.5%)	57 (24.7%)	18 (7.8%)	2 (0.9%)
Item 8	57 (24.7%)	42 (18.2%)	76 (32.9%)	45 (19.5%)	11 (4.8%)
**3 Option Items**					
	**No**		**Maybe**	**Yes**	
**Study group**					
Item 5	173 (62.5%)		78 (28.2%)	26 (9.4%)	
Item 6	190 (68.6%)		63 (22.7%)	24 (8.7%)	
Item 7	144 (52%)		102 (36.8%)	31 (11.2%)	
Item 9	65 (23.5%)		30 (10.8%)	182 (65.7%)	
**Control group**					
Item 5	163 (70.6%)		57 (24.7%)	11 (4.8%)	
Item 6	118 (51.1%)		32 (13.9%)	81 (35.1%)	
Item 7	111 (48.1%)		51 (22.1%)	69 (29.9%)	

No.—number; %—percentage.

**Table 4:** Correlations between age and Items 1, 3, and 8.

Correlations	*p* *
**Study group**	
Age (*p* < 0.001 **) x Item 1 Score (*p* < 0.001 **)	0.001, R = −0.204
Age (*p* < 0.001 **) x Item 3 Score (*p* < 0.001 **)	<0.001, R = −0.223
Age (*p* < 0.001 **) x Item 8 Score (*p* < 0.001 **)	0.001, R = 0.195
**Control group**	
Age (*p* < 0.001 **) x Item 1 Score (*p* < 0.001 **)	0.255, R = 0.075
Age (*p* < 0.001 **) x Item 3 Score (*p* < 0.001 **)	0.244, R = 0.077
Age (*p* < 0.001 **) x Item 8 Score (*p* < 0.001 **)	0.853, R = 0.012

* Spearman’s rho correlation coefficient, ** Shapiro–Wilk test.

**Table 5:** Patients’ distribution according to gender, living environment, and answers provided for different items.

**Gender/Answer (No., %)**	**Girls**	**Boys**	*p* *
**Study group**			
**Item 6**			
No	110 (63.6%)	80 (76.9%)	0.031
Maybe	48 (27.7%)	15 (14.4%)	
Yes	15 (8.7%)	9 (8.7%)	
**Item 7**			
No	76 (43.9%)	68 (65.4%)	0.002
Maybe	73 (42.2%)	29 (27.9%)	
Yes	24 (13.9%)	7 (6.7%)	
**Control group**			
**Item 6**			
No	73 (54.5%)	45 (46.4%)	0.376
Maybe	19 (14.2%)	13 (13.4%)	
Yes	42 (31.3%)	39 (40.2%)	
**Item 7**			
No	76 (56.7%)	35 (36.1%)	<0.001
Maybe	32 (23.9%)	19 (19.6%)	
Yes	26 (19.4%)	43 (44.3%)	
**Living Environment/Answer (No., %)**	**Rural**	**Urban**	***p*** *
**Study group**			
**Item 5**			
No	49 (52.7%)	124 (67.4%)	0.001
Maybe	39 (41.9%)	39 (21.2%)	
Yes	5 (5.4%)	21 (11.4%)	
**Item 7**			
No	59 (63.4%)	85 (46.2%)	0.005
Maybe	30 (32.3%)	72 (39.1%)	
Yes	4 (4.3%)	27 (14.7%)	
**Item 9**			
No	15 (16.1%)	50 (27.2%)	0.007
Maybe	17 (18.3%)	13 (7.1%)	
Yes	61 (65.6%)	121 (65.8%)	
**Control group**			
**Item 5**			
No	62 (67.4%)	101 (72.7%)	0.002
Maybe	30 (32.6%)	27 (19.4%)	
Yes	0 (0%)	11 (7.9%)	
**Item 7**			
No	47 (51.1%)	64 (46%)	0.065
Maybe	25 (27.2%)	26 (18.7%)	
Yes	20 (21.7%)	49 (35.3%)	

No.—number; %—percentage; * Fisher’s exact test.

**Table 6:** Patients’ distribution according to answers provided for Items 7 and 9.

Answer (No., %)	Correction neg.	Indecisive	Correction pos.	*p* *
Compulsoriness neg.	27 (41.5%)	22 (73.3%)	95 (52.2%)	0.020
Indecisive	25 (38.5%)	7 (23.3%)	70 (38.5%)	
Compulsoriness pos.	13 (20%)	1 (3.3%)	17 (9.3%)	

No.—number; %—percentage; neg.—negative; pos.—positive; * Fisher’s exact test.

**Table 7:** Comparison of answers provided for Items 2 and 4 in relation to gender.

Gender	Mean Value ± SD	Median (IQR)	Medium Rank	*p* *
**Study group**				
**Item 2**				
Girls (*p* < 0.001 **)	1.94 ± 1.07	2 (1–3)	147.58	0.012
Boys (*p* < 0.001 **)	1.71 ± 1.15	1 (1–2)	124.74	
**Item 4**				
Girls (*p* < 0.001 **)	2.86 ± 1.27	3 (2–4)	149.42	0.004
Boys (*p* < 0.001 **)	2.42 ± 1.41	2 (1–4)	121.66	
**Control group**				
**Item 2**				
Girls (*p* < 0.001 **)	3.05 ± 1.26	3 (2–4)	114.77	0.732
Boys (*p* < 0.001 **)	3.1 ± 1.29	3 (2–4)	117.70	
**Item 4**				
Girls (*p* < 0.001**)	2.04 ± 1.05	2 (1–3)	123.31	0.034
Boys (*p* < 0.001 **)	1.77 ± 1.06	1 (1–3)	105.90	

SD—standard deviation; IQR—interquartile range; * Mann–Whitney U test, ** Shapiro–Wilk test.

**Table 8:** Comparisons between various items.

Comparison	Answer	Mean Value ± SD	Median (IQR)	Medium Rank	*p* *
**Study group**					
Item 1 and Item 9	No (*p* < 0.001 **)	2.26 ± 1.35	2 (1–3)	157.08	0.014
	Maybe (*p* = 0.005 **)	1.67 ± 0.77	1.5 (1–2)	128.67	
	Yes (*p* < 0.001 **)	1.84 ± 1.19	1 (1–2)	129.55	
Item 2 and Item 5	No (*p* < 0.001 **)	1.98 ± 1.12	2 (1–2)	149.32	0.009
	Maybe (*p* < 0.001 **)	1.59 ± 0.98	1 (1–2)	119.13	
	Yes (*p* < 0.001 **)	1.85 ± 1.25	1 (1–3)	129.94	
Item 3 and Item 5	No (*p* < 0.001 **)	2.12 ± 1.34	2 (1–3)	132.34	0.025
	Maybe (*p* < 0.001 **)	2.33 ± 1.5	2 (1–4)	141.79	
	Yes (*p* < 0.001 **)	3.08 ± 1.69	3 (1–5)	174.94	
Item 3 and Item 7	No (*p* < 0.001 **)	2.51 ± 1.61	2 (1–4)	149.19	0.041
	Maybe (*p* < 0.001 **)	1.95 ± 1.18	1 (1–3)	124.51	
	Yes (*p* < 0.001 **)	2.16 ± 1.21	2 (1–3)	139.34	
**Control group**					
Item 3 and Item 5	No (*p* < 0.001 **)	2.5 ± 1.15	2 (2–4)	118.22	0.009
	Maybe (*p* < 0.001 **)	2.19 ± 1.1	2 (1–2)	100.78	
	Yes (*p* = 0.001 **)	3.27 ± 0.9	4 (2–4)	162.00	
Item 3 and Item 7	No (*p* < 0.001 **)	2.25 ± 1.08	2 (2–3)	104.69	0.007
	Maybe (*p* < 0.001 **)	2.41 ± 1.06	2 (2–4)	114.56	
	Yes (*p* < 0.001 **)	2.84 ± 1.23	3 (2–4)	135.26	

SD—standard deviation; IQR—interquartile range; * Kruskal–Wallis H test, ** Shapiro–Wilk test.

**Table 9:** Comparison of answers provided for Items 1, 2, 3, 4, and 8 according to the analyzed groups.

Groups	Mean Value ± SD	Median (IQR)	Medium Rank	*p* *
**Item 1**				
Control (*p* < 0.001 **)	2.42 ± 1.16	3 (1–3)	286.69	<0.001
Study (*p* < 0.001 **)	1.97 ± 1.25	2 (1–2)	227.65	
**Item 2**				
Control (*p* < 0.001 **)	3.07 ± 1.27	3 (2–4)	324.51	<0.001
Study (*p* < 0.001 **)	1.86 ± 1.1	1 (1–2)	196.11	
**Item 3**				
Control (*p* < 0.001 **)	2.46 ± 1.15	2 (2–4)	274.97	0.003
Test (*p* < 0.001 **)	2.27 ± 1.44	2 (1–4)	237.43	
**Item 4**				
Control (*p* < 0.001 **)	1.93 ± 1.06	1 (1–3)	209.61	<0.001
Test (*p* < 0.001 **)	2.69 ± 1.34	3 (2–4)	291.94	
**Item 8**				
Control (*p* < 0.001 **)	2.61 ± 1.18	3 (2–3)	300.39	<0.001
Test (*p* < 0.001 **)	1.92 ± 1.12	1 (1–3)	216.23	

SD—standard deviation; IQR—interquartile range; * Mann–Whitney U test, ** Shapiro–Wilk test.

**Table 10:** Comparison of answers provided for Items 5, 6, and 7 according to the analyzed groups.

Groups	No	Maybe	Yes	*p* *
**Item 5**				
Control	163 (48.5%)	57 (42.2%)	11 (29.7%)	0.064
Test	173 (51.5%)	78 (57.8%)	26 (70.3%)	
**Item 6**				
Control	118 (38.3%)	32 (33.7%)	81 (77.1%)	<0.001
Test	190 (61.7%)	63 (66.3%)	24 (22.9%)	
**Item 7**				
Control	111 (43.5%)	51 (33.3%)	69 (69%)	<0.001
Test	144 (56.5%)	102 (66.7%)	31 (31%)	

No.—number; %—percentage; * Fisher’s exact test.

## Data Availability

The data presented in this study are available on request from the corresponding authors. The data are not publicly available due to privacy reasons.
